# Bifunctional Peptides Generated by Optimising the Antimicrobial Activity of a Novel Trypsin-Inhibitory Peptide from *Odorrana schmackeri*

**DOI:** 10.3390/biom16010148

**Published:** 2026-01-14

**Authors:** Ying Wang, Xinchuan Chai, Ying Zhang, Xueying Xing, Yangyang Jiang, Tao Wang, Xiaoling Chen, Lei Wang, Mei Zhou, James F. Burrows, Na Li, Xiaofei Zhang, Tianbao Chen

**Affiliations:** 1 Shaanxi Province Key Laboratory of New Drugs and Chinese Medicine Foundation Research, School of Pharmacy, Shaanxi University of Chinese Medicine, Xi’an 712046, China; wangying011@sntcm.edu.cn (Y.W.); 524050401410@email.sntcm.edu.cn (X.C.); 522050101423@email.sntcm.edu.cn (Y.Z.); 522050101430@email.sntcm.edu.cn (X.X.); linachem@sntcm.edu.cn (N.L.); 2 Natural Drug Discovery Group, School of Pharmacy, Queen’s University Belfast, Belfast BT9 7BL, UK; yangyangjiang@qub.ac.uk (Y.J.); t.wang@qub.ac.uk (T.W.); x.chen@qub.ac.uk (X.C.); l.wang@qub.ac.uk (L.W.); m.zhou@qub.ac.uk (M.Z.); j.burrows@qub.ac.uk (J.F.B.)

**Keywords:** molecular cloning, Bowman–Birk-type trypsin inhibitors, bifunctional peptides, antibacterial activity, molecular dynamics simulations

## Abstract

Drug-resistant bacteria cause millions of global infections each year, and the development of alternative antimicrobial drugs has become a serious undertaking. Currently, peptides with antimicrobial activity represent potential candidates for new antibiotic discovery as they are less likely to cause drug resistance in bacteria. In this study, bifunctional peptides with potent trypsin-inhibitory activity and antimicrobial activity were obtained by rational computation-based structural modifications to a novel Bowman–Birk-type inhibitor (BBI) peptide. The analogues not only displayed potent bacterial killing ability against two drug-resistant bacteria strains of *E. coli* but also an excellent safety profile, as assessed by low haemolytic activity and low anti-proliferation activity on HaCaT cells. Throughout the molecular dynamics simulations, the peptides exhibited stable adsorption onto the mixed POPE/POPG membrane; most amino acid residues of the AMPs remained bound to the membrane surface, with a few amino acid residues partially penetrating the membrane interior. This showed that the electrostatic interactions were the dominant driving force mediating the peptide–membrane associations. In addition, the tested peptides displayed a degree of stability in the presence of salt ions, serum, and trypsin. These modified peptides thus possess potential as clinical antibacterial agents, and the strategies used in structural modification may also provide a different path to developing new antimicrobial peptides.

## 1. Introduction

Antibiotics are one of the greatest milestones in human medical history, but in recent decades, antimicrobial resistance (AMR) has been a growing threat to human health [1]. Poor prescriptive compliance and regulation of antibiotic use has led to antibiotic-resistant superbugs being generated, which have resulted in severe problems in both human medicine and veterinary medicine and in agriculture [2,3]. The rapid evolution of drug-resistant microorganisms and the development of antibiotic resistance have limited the effectiveness of conventional antibiotics [4,5]. The most recent survey of the Centers for Disease Control and Prevention (CDC) indicates that, each year, millions of patients are infected with antibiotic-resistant bacteria globally, and it is estimated that the number of AMR-related deaths will reach tens of millions by 2050. Therefore, it has become a matter of urgency to develop novel efficacious antimicrobial agents [6].

In recent years, numerous functional components from amphibian-derived skin secretions have been studied [7,8,9]. Amphibian skin secretions have long been known to be a unique source of bioactive compounds, including antimicrobial peptides (AMPs). Peptides with antimicrobial activity are potential candidates for new antibiotic discovery, as they are thought to be less likely to cause drug resistance in bacteria [10]. AMPs with positive charges, by interacting with the cationic cell membranes of microorganisms, can cause cell death by binding to and compromising bacterial membranes [11]. This mechanism is different to that of conventional antibiotics, as the targets AMPs can bind to are situated on the cell membrane surface, so microbes can be killed in a short time by such electrostatic interactions [12]. This renders induction of resistance to AMPs a low possibility [13]. However, non-selective mammalian cytotoxicity and haemolytic activity can be observed for some AMPs which have low selectivity for bacterial cell membranes [14]. Another drawback of AMPs is that they regularly possess positive charges donated by lysine and arginine, and as a result, these AMPs may be readily degraded by trypsin or trypsin-like proteases [15]. Previous research reported that the structurally related BBI peptide OSTI [16] exhibited potent trypsin-inhibitory activity. In contrast, numerous trypsin-inhibitory peptides are known to possess both strong trypsin-inhibitory and low antimicrobial activities, along with minimal cytotoxicity and haemolysis [7,17].

In this study, OSTI-1872, a novel peptide of the Bowman–Birk type and predicted to be a trypsin inhibitor, was identified in the skin secretion of *Schmacker*’s frog, *Odorrana schmackeri*. The peptide showed strong inhibitory activity against trypsin and weak antimicrobial activities. Therefore, several analogues were strategically designed to optimise its antimicrobial activity while retaining its inhibitory activity. Enhanced antimicrobial activity and potent inhibitory activity were demonstrated by the analogue peptides OSTI-2369, OSTI-2483, and OSTI-2380. These analogues also showed excellent results regarding their safety profiles, with low haemolytic activity and low activity against HaCaT cell proliferation. In addition, the analogues maintained their antimicrobial activity upon treatment with trypsin for 1 h. Molecular dynamics simulations further revealed that the binding of these bifunctional peptides to the bacterial membrane surface was predominantly driven by electrostatic interactions. In conclusion, these bifunctional peptides are worthy of further study and may be potential candidates as novel clinical antibacterial agents.

## 2. Materials and Methods

### 2.1. Acquiring Skin Secretion of Schmacker’s Frog, Odorrana schmackeri

Four adult specimens of *Odorrana schmackeri* were collected in Fujian Province, China. Then, the adult specimens were used to collect skin secretions by gentle electrical stimulation for 30 s (5 V; 3 ms pulses) on the dorsal skin using a bipolar electrode moving around the skin surface of the frog (C.F. palmer, London, UK). Afterwards, the secretion on the skin was washed off with double-distilled water (ddH_2_O), swiftly lyophilised by freezing in liquid nitrogen and then placed into a 1–2/LD freeze dryer (Martin Christ, Osterode am Harz, Germany) and finally stored at −20 °C.

### 2.2. Molecular Cloning of Skin Peptides

The lyophilised skin secretion of *Odorrana schmackeri* (5 mg) was weighed and 1 mL of lysis/binding buffer was used to dissolve it in an RNase-free tube. After vortexing and centrifugation, the supernatant in the tube was transported into another tube containing Dynabeads^TM^ Oligo (dT)25 (Thermo Fisher Scientific Baltics USB, Vilnius, Lithuania). Then, after being treated with Tris-HCl (pH 7.5), the mixture was heated at 80 °C for 2 min to collect mRNA. The Invitrogen^TM^ SuperScript^TM^ III Reverse Transcriptase (Thermo Fisher Scientific, Waltham, MA, USA) was used to synthesise the first-strand cDNA. The 3′ RACE-Ready cDNA was generated using a Clontech SMARTer^@^ RACE 5′/3′ Kit (TaKaRa, Shiga, Japan), which contained a Nested Universal Primer (NUP) and a designed degenerate sense primer (S: 5′-GCIGCIYTIAARGGITGYT-3′). The designed primer was chosen as it was complementary to the amino acid sequence of ‘A-A-L/I-K-G-C-W-’ at the N-terminal of skin peptides isolated from frogs. A 3′-Rapid Amplification of cDNA Ends (RACE) PCR was conducted to construct a cDNA library by using a Clontech Advantage^@^ 2 PCR Kit (TaKaRa, Dalian, China) and a polymerase chain reaction (PCR) machine (ThermoFisher Scientific, Waltham, MA, USA). The PCR programme was set as follows: 1 min for initial denaturation at 94 °C; 40 cycles for further denaturation (94 °C, 30 s), primer annealing (59 °C, 30 s) and extension (72 °C, 3 min); and finally, another 10 min at 72 °C for the final extension. Gel electrophoresis was chosen to analyse the PCR product. The purified product was obtained by an E.Z.N.A.^@^ Cycle Pure Kit (Omega BioTek, Norcross, GA, USA). The target DNA was selected via the ligation process by using a pGEM@-T Easy Vector Systems (Promega, Madison, WI, USA), which contains the gene of interest, and was cloned in JM109 high-efficiency competent cells (Promega, Madison, WI, USA). After collection of the DNA product, it was cloned by PCR, analysed, and purified again as described above. Finally, the sample was processed by using 95% ethanol and 70% ethanol; then sequence analysis and identification were carried out by the Genomic CTU of the university.

### 2.3. Bioinformatic Analysis

The sequence of nucleotides was translated to amino acids through use of the online programme Expasy Translate (https://web.expasy.org/translate/ (accessed on 10 December 2025)). The sequence alignments of the translated open reading frame were accomplished using the web programme NCBI-BLAST (https://blast.ncbi.nlm.nih.gov (accessed on 10 December 2025)).

### 2.4. Peptide Design and Solid-Phase Peptide Synthesis (SPPS)

The analogues of the parent peptide were modified strategically in terms of aspects including cationic charge, hydrophilicity, and secondary structure, employing the artificial secondary structures of the α-helix and ß-sheet referred to in the reported literature [9,18,19,20]. The automated solid-phase peptide synthesiser was used to synthesise the parent peptide and analogues (Protein Technologies, Tucson, AZ, USA). Each amino acid of the peptide was weighed into a vial, and 2-(1H-benzotriazole-1-yl)-1,1,3,3-tetramethyluronium hexafluorophosphate (HBTU, a coupling reagent) was also weighed and mixed with each amino acid. The products were cleaved from the resin by a mixture solution comprising 94% trifluoroacetic acid (TFA), 2% ddH_2_O, 2% thioanisole (TIS), and 2% 1, 2-ethanedithiol (EDT). Then, the synthetic peptides were lyophilised and stored at −20 °C.

### 2.5. Prediction of Physicochemical Properties and Secondary Structure

The peptides’ physicochemical properties were computed using the online proteomic bioinformatic resource BACHEM (https://www.bachem.com/knowledge-center/peptide-calculator/ (accessed on 10 December 2025)) and the HeliQuest server (https://heliquest.ipmc.cnrs.fr/cgi-bin/ComputParams.py (accessed on 10 December 2025)). The secondary structure prediction of the peptides was performed by using the online AlphaFold Server (https://alphafoldserver.com).

### 2.6. Purification and Identification of Peptides

Reverse-phase High-Performance Liquid Chromatography (RP-HPLC) was used to purify crude synthetic peptides based on their different polarity or/and hydrophobicity via a LUNA C-5 preparation chromatographic column (250 × 10 mm, Phenomenex, Torrance, CA, USA), which acts as stationary phase in this system. The mobile phase contains two solutions. Solution A is ddH_2_O with 0.5% TFA. Solution B involves 80% acetonitrile, 19.5% ddH_2_O with 0.5% TFA.

Matrix-assisted laser desorption ionisation–time of flight mass spectrometry (MALDI-TOF MS) (Voyager DE, Perseptive BioSytems, Framintham, MA, USA) was used to identify synthetic peptides. The solvent of CHCA (10 mg/mL), consisting of 70% acetonitrile and 30% water with 0.1% TFA, was used as a matrix solution for peptide analysis. A total of 2 μL of HPLC fractions and 1 μL of CHCA solution were loaded in the plate. The mass/charge ratios (M/Z) were recorded to identify the target peptide.

### 2.7. Circular Dichroism

The secondary structures of peptides were investigated using a JASCO-815 circular dichroism (CD) spectrometer (Jasco, Essex, UK). Peptides were prepared at a stock concentration of 100 µM with 20 mM NH4Ac solution; then, stock peptide solutions were mixed at ratio 1:1 with ddH_2_O and TFE to working solutions. The measurement was conducted at 20 °C; the selective wavelength was 190–260 nm, with a 1 nm bandwidth, 0.5 nm data pitch, and a scanning speed of 100 nm/min. The CD data were analysed with the online tool K2D3 (http://cbdm-01.zdv.uni-mainz.de/~andrade/k2d3/ (accessed on 10 December 2025)).

### 2.8. Trypsin Inhibition Determination

The substrate of trypsin in this assay was Phe-Pro-Arg-AMC (Bachem, Merseyside, UK). The peptide was dissolved in phosphate-buffered saline (PBS) to a series of concentrations (1–1000 μM); it was then added to a black 96-well plate with 180 μL of substrate (50 μM in PBS) and 10 μL of trypsin (0.1 μM in 1 mM HCl) under dark conditions. Each well of the black plate contained a final volume of 210 μL. Then, the plate was measured immediately after trypsin was added, and the fluorescence intensity of each well was recorded for 30 min every 30 s via a FLUOstar OPTIMA plate reader (BMG Labtech, Ortenberg, Germany). The emission and excitation of wavelengths of 460 nm and 395 nm, respectively, were used, and the temperature was set to 37 °C. The curves of trypsin-inhibitory activity were constructed by using the Morrison equation in Prism 9 (working concentration of substrate, [S] = 42.86 μM; working concentration of trypsin, Et = 0.0020 μM; trypsin kinetic constant, Km = 41.07 μM).

### 2.9. Antimicrobial Activity Determination Assays

Minimum inhibitory concentration (MIC) values represent peptides at the lowest concentrations that can inhibit visible bacterial growth. Minimum bactericidal concentration (MBC) values indicate peptides at the lowest concentrations that can kill all bacteria.

Ten microorganisms were selected for the antimicrobial assay, which were *Escherichia coli* (*E. coli*, ATCC CRM 8739) (*E. coli*, BAA 2340) (*E. coli*, NCTC 13846), *Pseudomonas aeruginosa* (*P. aeruginosa*, ATCC CRM 9027), *Staphylococcus aureus* (*S. aureus*, ATCC CRM 6538), *Candida albicans* (*C. albicans*, ATCC 10231), *Enterococcus faecium* (*E. faecium*, NCTC 12697), Methicillin-resistant *Staphylococcus aureus* (MRSA, NCTC 12493), *Klebsiella pneumoniae* (*K. pneumoniae*, ATCC CRM 43861), and *Acinetobacter baumannii* (*A. baumannii*, BAA 747). The culture media for bacteria were tryptic soy broth (TSB), tryptic soy agar (TSA), nutrient broth (NB), and nutrient agar (NA). The culture media for yeast were yeast extract peptone dextrose broth (YPD-B) and yeast extract peptone dextrose agar (YPD-A).

A sample of the stocks of microorganisms from a −20 °C freezer was separately inoculated into flasks with appropriate culture media, after which the flasks were placed in an Orbital Shaker for 16–20 h at 120 rpm/min, 37 °C, or 26 °C for yeast. Then, 0.5 mL of the cultures was transferred in McCartney bottles with 20 mL of broth media to be sub-cultured under the same conditions. When the optical densities (ODs) reached appropriate values at a wavelength of 550 nm, the sub-cultures were diluted 200 times using the same fresh media to achieve a working cell density of 5 × 10^5^ CFU/mL. Dimethyl sulfoxide (DMSO) was chosen to dissolve peptides at concentrations ranging from 100 μM to 51,200 μM. Then, different media were loaded into a 96-well plate. Experimental groups comprised 99 μL diluted culture and 1 μL peptide solution. The vehicle control contained 99 μL diluted culture and 1 μL DMSO. The positive control contained 1 μL norfloxacin (for bacteria, 20 μg/mL) or 1 μL of amphotericin B (for yeast, 10 μg/mL) and 99 μL of diluted culture. A volume of 100 μL of sterile broth medium acted as the negative control, and 100 μL of the diluted culture was used as the growth control. The loaded 96-well plate was incubated for 20–24 h, and the OD value was obtained at 550 nm through the use of a plate reader—Synergy HT (BioTek, Winooski, VT, USA).

The minimal concentration of the experimental group with no visible bacterial growth was identified as the MIC. Cultures in the experimental groups were inoculated on the plate with the corresponding solid culture medium and incubated under the same conditions as for MIC determination. The values of MBCs were the lowest concentrations of peptide without organism growth.

### 2.10. Time-Killing Assays

The time-killing assay was designed to define the kinetic killing ability of peptides against bacteria. The selected strains used in this assay were drug-resistant *Escherichia coli* (*E. coli*, NCTC 13846) and (*E. coli*, BAA 2340). The bacterial strains were cultured using the same method as that employed in the antimicrobial activity determination assay. Then, 198 μL of the bacterial suspension (5 × 10^5^ CFU/mL) was treated with 2 μL of peptide solutions with the concentrations of MIC, 2 × MIC and 4 × MIC in 3 sterile tubes. The bacteria–peptide mixture medium was diluted 10, 100, and 1000 times, and 10 μL of mixture medium at four different concentrations was seeded onto plates with solid culture medium NA at time points of 0, 5, 10, 15, 30, 60, 90, 120 and 180 min. All colonies were counted after seeded plates were incubated at 37 °C for 24 h. The growth control with no treatments was 200 μL of bacteria medium, and the vehicle control consisted of 198 μL bacteria medium treated with 2 μL DMSO.

### 2.11. SYTOX Green Permeability Assays

SYTOXTM Green Nucleic Acid Stain (ThermoFisher Scientific, Waltham, MA, USA) will not cross intact membranes but can easily penetrate compromised membranes characteristic of dead cells, making it a valuable indicator of dead bacteria.

Bacteria (*E. coli*, NCTC 13846/*E. coli*, BAA 2340) were cultured with TSB in an Orbital Shaker at 37 °C overnight. After being sub-cultured for 2 h, the bacteria were centrifuged for 10 min at 4 °C and 1000× *g*. Then, the culture medium was discarded, and the bacteria were washed twice with 5% TSB (in 0.85% NaCl). The density of washed bacteria reached the logarithmic growth phase when the OD value reached 0.70 at a detected wavelength of 590 nm in the 5% TSB solution. Next, 40 μL of peptide solutions at concentrations of respective 2.5 × MIC, 5 × MIC, and 10 × MIC and 50 μL of bacterial suspension were loaded into a black 96-well plate and then incubated at 37 °C for 2 h. Afterwards, 10 μL of SYTOX^TM^ green nucleic acid stain (5 μM) was added into the plate, and the mixture was incubated without light for 5 min at 37 °C. The final peptide concentration in the 96-well plate was MIC, 2 × MIC, 4 × MIC. The intensity of fluorescence was determined using a Synergy HT plate reader (BioTech, Winooski, VT, USA). The wavelengths of excitation and emission were set at 485 and 528 nm, respectively. Bacterial medium with 5% TSB was used as the negative control; bacterial medium with Melittin peptide solution (8 μM) acted as the positive control; 5% TSB was used as the blank control.

### 2.12. Safety Evaluation

#### 2.12.1. Haemolysis Assays

The purpose of the haemolysis assay was to assess the lytic activity of peptides against horse red blood cells in vitro. The erythrocytes were acquired from defibrinated horse blood and washed with PBS to reach a 4% suspension in PBS. The peptide was dissolved in DMSO to make a stock solution and then diluted by PBS to working concentrations ranging from 2 μM to 1024 μM (DMSO was less than 1% in total volume). Next, 100 μL of peptide solution and 100 μL of 4% erythrocyte suspension were added into 36 tubes and were then incubated at 37 °C for 2 h. Afterwards, these tubes were centrifuged for 10 min at 930× *g* and the supernatant in each tube was carefully transferred into a 96-well plate. The lysis of erythrocytes in the plate was detected at 470 nm by using the Synergy HT plate reader (BioTek, USA). A 4% erythrocyte suspension treated with 1% Triton X-100 (Sigma-Aldrich, St. Louis, MO, USA) acted as the positive control, and the negative control consisted of 4% erythrocyte suspension and 1% DMSO solution (diluted by PBS).

#### 2.12.2. MTT Cell Antiproliferation Assays

The MTT assay is used for the safety evaluation of the antiproliferative activity of a peptide against normal cells. HaCaT cells, which are a keratinocyte cell line from adult human skin, were purchased from the American Type Culture Collection (ATCC, Manassas, VA, USA).

The revived cells were cultured for 3–5 days at 37 °C until they were attached to the flask wall. After this, the medium was removed, and the wall was washed gently twice with 10 mL of PBS and similarly removed. Next, 3–4 mL of trypsin solution was used to detach the cells from the container wall, and then 8–10 mL FBS was added to the flask to stop the digestion reaction. The cell suspension was transported into a 15 mL tube and centrifuged for 6 min at 130× *g*, 18 °C. The supernatant in the tube was removed and 4 mL of complete growth medium was added to make a stock cell suspension. The mixture, containing an equal volume of trypan blue and cell medium, was transferred into a chamber to measure the density of cancer cells by use of a microscope. The cell medium was diluted by foetal bovine serum (FBS) at a standard cell density of 1 × 10^5^ cells/mL based on the cell density. Then, 100 µL of cell suspension was transferred into each well of a 96-well plate. After 20–24 h incubation, 100 μL of the serum-free medium was used as the new culture medium to substitute the previous FBS. The cells were starved in an incubator for 4 h. The peptide was prepared at the working concentrations ranging from 10^−9^ to 10^−4^ M with serum-free medium. The sample group contained 100 μL of working concentration peptide solution. The vehicle group contained 1 µL DMSO and 99 µL serum-free medium. Blank and growth groups contained 100 µL of serum-free medium. After treatment, the loaded 96-well plate was incubated for another 24 h. Then, 10 µL of MTT was added to each well and incubated for 2 h. Finally, the medium in each well was discarded and replaced with 100 μL of DMSO. After 10 min shaking, the cell viability in each well was detected at 570 nm (BioTek, USA).

### 2.13. Factors Impacting Stability of Antimicrobial Activity

#### 2.13.1. Salt Ions and Serum Sensitivity

The sensitivity of the peptides to salt ions and serum was examined in the antimicrobial activity determination assay in the presence of such. *E. coli* (ATCC CRM 8739) was the tested bacterium. Different concentrations of salts (150 mM NaCl, 4.5 mM KCl, 6 µM NH_4_Cl, 1 mM MgCl_2_, 2.5 mM CaCl_2_, and 4 mM FeCl_3_) were used to determine the influence of cationic substances in the bacterial culture towards the antimicrobial activities of the peptides.

#### 2.13.2. Trypsin Sensitivity

The peptide was dissolved with PBS to a stock concentration of 12,800 μM, and the concentration of the trypsin solution was set at 0.25 mg/mL and 0.5 mg/mL. (The molecular weight of trypsin is about 24 K, and the converted molar concentrations are about 10,417 μM and 20,833 μM, which is close to the peptide stock concentration and 2-fold higher, respectively.) Next, equal volumes of trypsin solution and peptide solution were added to a tube and then incubated in a dry block heater at 37 °C for 1 h and then up to 60 °C for 0.5 h to inactivate the trypsin. The control group of peptide solution was incubated along with the mixture. The following procedures were as the same as described for the antimicrobial activity determination assay mentioned before.

### 2.14. Molecular Dynamics Simulations of the Interactions Between the Peptide and the Anionic Lipid Membrane

Molecular dynamics simulations were conducted on three peptides with distinct structural features from the parent peptide and its analogues to compare their interaction patterns with the computer-simulated bacterial cell membrane.

The cell membrane was built using CHARMM-GUI, consisting of POPE and POPG lipids in a molar ratio of 3:1. The membrane patch measured 10.006 × 10.006 nm^2^ and contained a total of 252 POPE and 84 POPG molecules. The C-termini of the peptides were amidated. The predicted peptide structures were visualised and verified using PyMOL 2.5.5, and the model with the highest prediction score was selected for molecular dynamic (MD) simulations.

The peptide was initially placed above the cell membrane. Water molecules were subsequently added to solvate the system, and appropriate amounts of Na^+^ and Cl^−^ ions were introduced to neutralise the net charge and to reach a physiological ionic strength of 0.15 M. The total number of atoms in the three simulation systems was 121,928, 126,056, and 127,391, respectively.

The CHARMM36 force field was employed to parameterize the peptides and the POPE and POPG lipids, while the TIP3P model was applied for water molecules. The force field parameter files for the peptides, POPE, and POPG molecules were generated using the pdb2gmx module in GROMACS 2023.3. After energy minimization and pre-equilibration under the NVT and NPT ensembles, MD simulations were carried out for 500 ns. Particle-mesh Ewald was used to treat electrostatic interactions. Trajectory snapshots were obtained using VMD. The time step in the production run was set to 2 fs, and coordinate frames were saved every 10 ps. Periodic boundary conditions were applied to all simulations.

### 2.15. Statistical Analysis

Statistical analysis of biological activity determination assays was conducted using Prism 9 (GraphPad software, Boston, MA, USA). One-way/two-way ANOVA was used for the analysis of the statistical significance of the differences. The data points are the mean of the independent experiments, and the error bar represents the standard error of the mean (SEM). Ns represents non-significance difference; * represents *p* < 0.05, ** represents 0.001 < *p* < 0.01, *** represents 0.0001 < *p* < 0.001, and **** represents *p* < 0.0001.

## 3. Results

### 3.1. The Discovery of a Novel Bowman–Birk-Type Inhibitor Peptide

**Figure 1:**
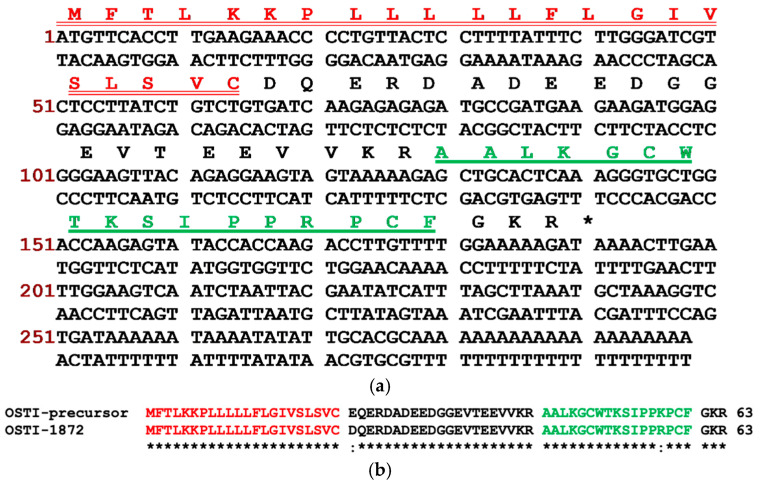
(**a**) The precursor-encoding cDNA sequence and its translated amino acid sequence. The residues of the putative signal peptide are double-underlined in red, and the residues of the mature peptide are single-underlined in green. The asterisk indicates the termination codon. (**b**) Alignment of respective peptide precursors. The asterisks indicate identical amino acid residues.

The precursor cDNA with a complete nucleotide sequence (Figure 1a) of a Bowman–Birk-type inhibitor (BBI) peptide was cloned via molecular cloning and contained 198 base pairs encoding 63 amino acids. The translated open reading frame comprised a putative 22-amino acid residue signal peptide, a 21-amino acid residue acidic spacer peptide, and a 17-amino acid residue mature peptide region. The mature peptide, named OSTI-1872, terminated in a Glycine residue which acted as an amide donor for C-terminal amidation. The ‘-KR-’ residues are a characteristic site for propeptide convertase cleavage. The peptide alignments were created using NCBI-BLAST (https://www.ncbi.nlm.nih.gov/ (accessed on 10 December 2025)). The results revealed a high degree of similarity between the novel peptide precursor and the OSTI precursor, both of which belong to the trypsin-inhibitory peptide group of the BBI family (Figure 1b). The cDNA sequence has been deposited in the Genbank Nucleotide Sequence Database under the accession number OR902190.

### 3.2. Structural Modification and Physicochemical Property Analysis

**Figure 2:**
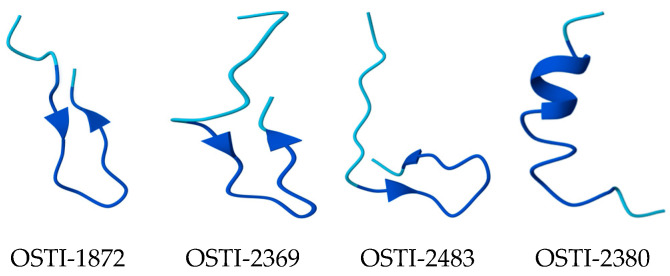
The secondary structure of peptides predicted by AlphaFold Server. In the figure, blue indicates pLDDT > 90, while cyan represents 70 < pLDDT ≤ 90. The pLDDT score is an atom-based confidence metric ranging from 0 to 100, with higher values reflecting greater prediction reliability.

In order to optimise the antimicrobial activity of OSTI-1872 while retaining its inhibitory activity, several structural modifications were proposed with regard to cationic charge, amphiphilicity (Table 1), and secondary structure (Figure 2) to improve the antimicrobial activity of these trypsin inhibitors using characteristics of antimicrobial peptides. The disulfide bridge of BBI peptides is a highly conserved hendecapeptide loop, called the trypsin-inhibitory loop (TIL). OSTI-2369 was created by adding an artificial α-helix structure to the N-terminus of the TIL of OSTO-1872 to increase its amphipathicity. For OSTI-2483, an artificial β sheet structure was added to the N-terminal of the TIL of the parent peptide, and three lysines were used to substitute for other amino acids to enhance the positive charge of peptides at the flexible 11, 14, and 17 positions. OSTI-2380 was designed to remove, or decrease, the trypsin-inhibitory activity of this peptide by disulfide bridge fracture to confirm the changes in its antimicrobial activity.

### 3.3. Circular Dichroism (CD) Spectroscopy

**Figure 3:**
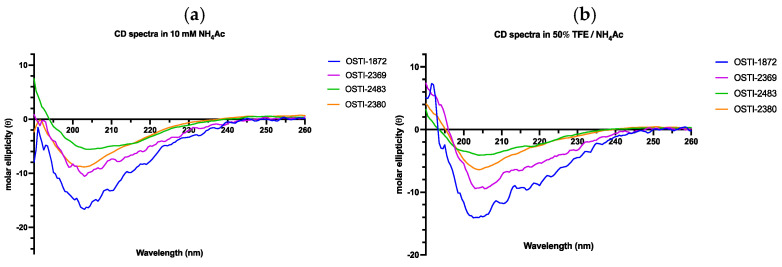
The CD spectra of OSTI-1872 and its analogues were detected in two different environments: (**a**) a 10 mM NH_4_Ac buffer acts as an aqueous environment; (**b**) a 50% TFE/NH_4_Ac solution is a microbial membrane-mimetic environment.

After the synthetic peptides were identified by MALDI-TOF MS (Figure S1) and the purity was analysed by Rp-HPLC (Figure S2), the precise secondary structures of OSTI-1872 and its analogues were determined by circular dichroism (CD) spectroscopy in an aqueous environment, which is a solution containing 10 mM ammonium acetate (NH_4_Ac), and a membrane-mimetic solution composed of 50% (*v*/*v*) trifluoroethanol (TFE) in 10 mM NH_4_Ac (Figure 3). In general, all peptides displayed a mixed conformation of α-helical, β-strand, and random coil, either in aqueous or membrane-mimetic environments. In addition, OSTI-2483 and OSTI-2380 possess a bigger proportion of β-strand than OSTI-1872 and OSTI-2369, as expected. What is more, it appeared that the proportion of α-helical conformations of OSTI-1872 and OSTI-2369 experienced a greater increase in a membrane-mimetic environment than in an aqueous one.

### 3.4. Inhibition Activity

**Figure 4:**
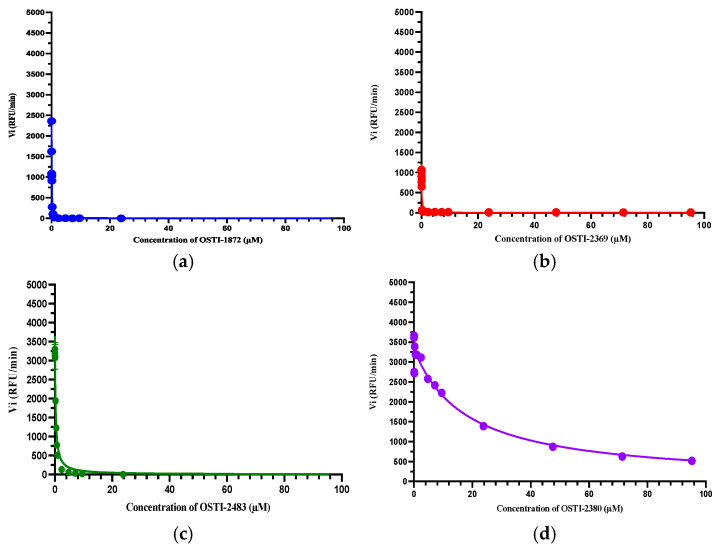
The Morrison inhibition curves of (**a**) OSTI-1872, (**b**) OSTI-2369, (**c**) OSTI-2483, and (**d**) OSTI-2380. The Ki values were calculated by the Morrison formula in Prism 9, in which trypsin Km = 41.07 μM, [S] = 42.86 μM, Et = 0.0020 μM.

The inhibitory activity of these peptides against trypsin was detected by a functional assay. OSTI-1872 exhibited potent trypsin-inhibitory activity with a Ki value of 0.023 μM. As expected, the trypsin-inhibitory activity of the modified peptides was retained at the same level compared to the parent peptide, except OSTI-2380, which experienced a sharp drop to a Ki of 8.779 μM, as its disulfide bridge was designed to break down (Table 2). The Morrison inhibition plots of tested peptides are shown in Figure 4.

### 3.5. Antimicrobial Activity of OSTI-1872 and Its Analogues

Antibacterial activities of the synthesised peptides in vitro were estimated by MIC/MBC determination assays with a representative set of microorganisms. Generally, the analogues displayed greatly increased antimicrobial activity against Gram-negative bacteria, including two drug-resistant strains of *E. coli*. In addition, the enhancement was also observed in analogues against a Gram-positive strain of MRSA. However, the analogues showed no improvement in antimicrobial activity against Gram-negative strains of *A. baumannii*, Gram-positive strains of *S. aureus* and *E. faecium*, and yeast. The analogues OSTI-2483 and OSTI-2380, conjugated with an artificial β-strand structure, enjoyed a much better bacterial killing ability in contrast to the parent peptide OSTI-1872. It seems that the anti-trypsin activity of these bifunctional peptides did not play a key role in their antimicrobial activity, as there was no obvious difference observed between OSTI-2483 and OSTI-2380. The results are presented in Table 3.

### 3.6. SYTOX Green Permeability

**Figure 5:**
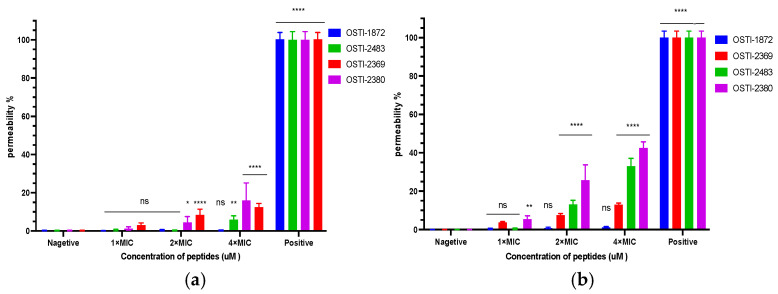
(**a**) The membrane permeability of peptides against *E. coli* NCTC 13846. (**b**) The membrane permeability of peptides against *E. coli* BAA 2340. The concentrations of all tested peptides were set at 1 × MIC, 2 × MIC and 4 × MIC. The positive and negative controls were bacteria treated with 8 μM Melittin and 5% TSB, respectively. The error bar is the SEM, which contains six replicates from two independent experiments, and the results of significant differences were calculated by comparing the negative control and the sample control.

The cell membrane permeability of these peptides was studied by a SYTOX green permeability test (Figure 5). The peptides were used at concentrations of MIC, 2 × MIC, and 4 × MIC against *E. coli* (NCTC 13846 and BAA 2340). In general, the experimental results demonstrated that all tested peptides produced partial membrane rupture of the two *E. coli* strains after 2 h of incubation. Meanwhile, the permeability of these *E. coli* strains caused by the peptides was low compared with the positive control (Melittin), with values less than 10% at the concentration of MICs. In particular, the analogue OSTI-2380, without a disulfide bridge, had the maximum instantaneous permeability effects.

### 3.7. Kinetic Time-Killing Ability

**Figure 6:**
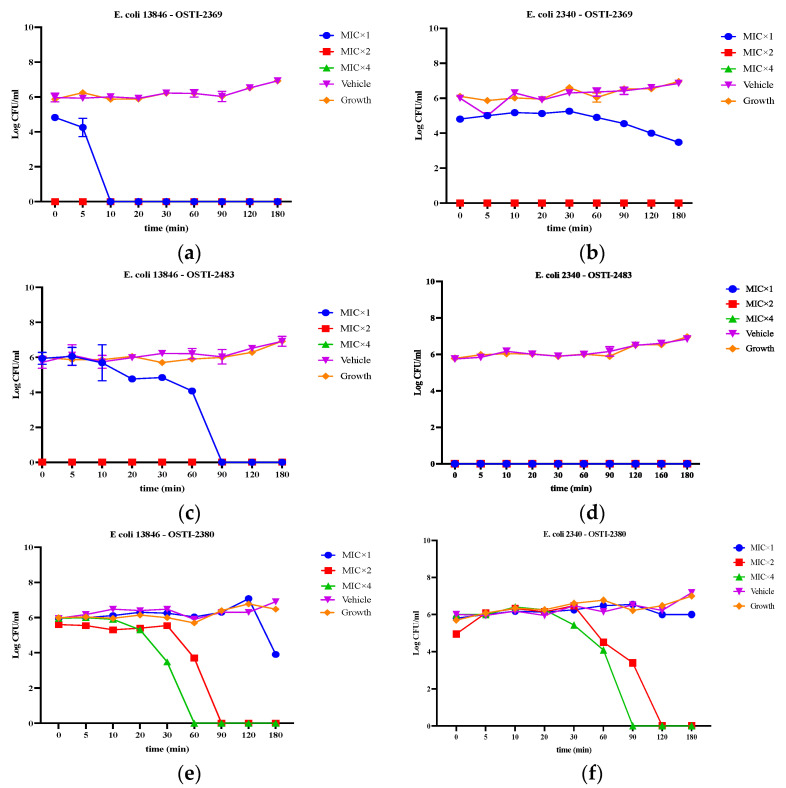
The kinetic time-killing curves of OSTI-2369 against *E. coli* 13846 (**a**) and *E. coli* 2340 (**b**), OSTI-2483 against *E. coli* 13846 (**c**) and *E. coli* 2340 (**d**), and of OSTI-2380 against *E. coli* 13846 (**e**) and *E. coli* 2340 (**f**). The blue, red, and green represent the peptide solution at concentrations of MIC, 2 × MIC, and 4 × MIC, respectively. Growth controls are in orange with no treatments; vehicle controls representing 1% DMSO-treated bacteria are purple. The *Y*-axis is the log CFU/mL for all groups and the *X*-axis represents time from 0 to 180 min. The error bar represents the SEM of six replicates in two independent experiments.

The time-killing assay was studied to understand the bactericidal kinetics of these peptides on *E. coli* strains NCTC 13846 and BAA 2340 (Figure 6). The concentrations of peptides used in this test were 1 × MIC, 2 × MIC, and 4 × MIC. The results showed that OSTI-2369 could kill *E. coli* strain NCTC 13846 within 10 min but did not show bactericidal activity against *E. coli* strain BAA 2340 within 360 min; OSTI-2483 killed *E. coli* strain NCTC 13846 within 90 min and *E. coli* strain BAA 2340 almost immediately at MIC. It is interesting that OSTI-2369 and OSTI-2483 both had instant bactericidal activity against the two tested strains at concentrations of 2 × MIC and 4 × MIC. OSTI-2380, which has lower anti-trypsin activity due to its disulfide bridge cleavage, had much slower bactericidal kinetics at the tested concentrations.

### 3.8. Safety Evaluation

**Figure 7:**
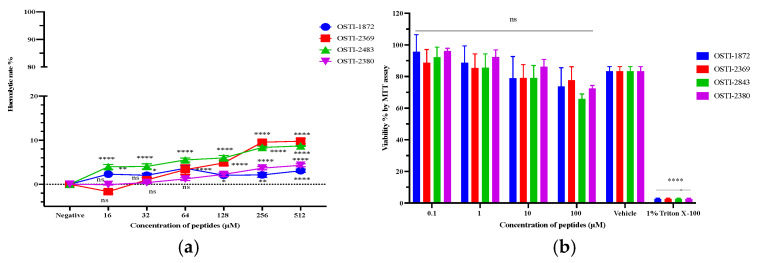
(**a**) The haemolytic activity of OSTI-1872 and analogues at concentrations of 1–512 μM against horse erythrocytes. The haemolytic percentage was calculated by comparing with the effect of the positive control and negative control, which were 1%Triton X-100 and PBS, respectively. (**b**) The antiproliferation activity of OSTI-1872 and analogues against HaCaT cells. 1% Triton X-100 and 1% DMSO (in PBS) acted as positive control and vehicle control, respectively. The error bar represented the standard error of the mean (SEM) with nine replicates in three separate experiments.

It is essential to study the cytotoxicity of these peptides to make a comprehensive evaluation of their potential. The haemolytic activity of the four peptides was evaluated using horse erythrocytes in vitro (Figure 7a). Based on these data, it was clear that both the parent peptide and its analogues produced little haemolysis, with less than 10% even at the highest concentrations. In terms of the cytotoxicity of OSTI-1872 and analogues towards the normal human cell line, HaCaT, the results (Figure 7b) of the MTT cell proliferation assay showed that the tested peptides had a minimal impact at concentrations between 0.1 and 10 μM but did reduce proliferation by more than 20% at 100 μM.

### 3.9. Factors Possibly Affecting the Stability of Antimicrobial Activity

#### 3.9.1. Salt Ions and Serum Effects

The stability of peptide effects against bacteria was studied in the presence of diverse salt ions and in horse serum. The results (Table 4) showed that all tested peptides maintained a degree of stable antibacterial effects when treated with horse serum and salt ions, except CaCl_2_, in whose presence all peptides experienced a decrease, especially peptide OSTI-2369, whose activity was reduced dramatically. The analogue of OSTI-2483 with β-strand and trypsin-inhibitory activity showed the best stability.

#### 3.9.2. Trypsin Stability

After incubation with trypsin, peptides were tested to determine whether their antimicrobial activity had been retained. It was found that all the analogues possessed a degree of resistance to degradation by trypsin, except OSTI-2380, whose trypsin-inhibitory activity decreased due to the disulfide bridge fracture. The MIC/MBC values of OSTI-2483 and OSTI-2380 indicate that the antimicrobial ability of analogues was related to their anti-trypsin inhibitory activity (Table 5).

### 3.10. Molecular Dynamics Simulation

#### 3.10.1. Representative Snapshots of the Molecular Dynamics Process

**Figure 8:**
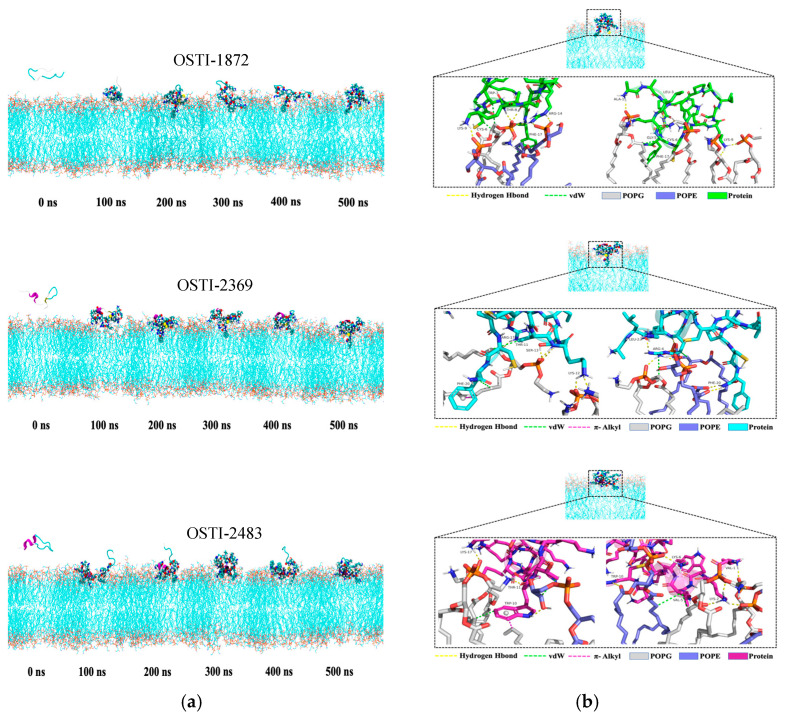
(**a**) Representative molecular dynamics snapshots; the peptide is shown in cartoon mode, the mixed-cell membrane is shown in line mode, and the peptide amino acid residues within 4 Å of the cell membrane are shown in VDW mode. (**b**) Details of the interaction between the peptide and mixed-cell membranes during molecular dynamics. In figures, red, blue, and orange denote oxygen, nitrogen, and carbon atoms, respectively.

Figure 8 shows a representative snapshot of molecular dynamics. During the dynamics, the three peptides stably adsorbed onto the POPE and POPG mixed-cell membrane, and some amino acid residues of each peptide were embedded in the cell membrane, indicating that three peptides can form stable associations with the mixed-cell membrane. However, the adsorption and embedding patterns differed, indicating that the interactions between peptides and the mixed-cell membrane are different.

#### 3.10.2. RMSF and RMSD

**Figure 9:**
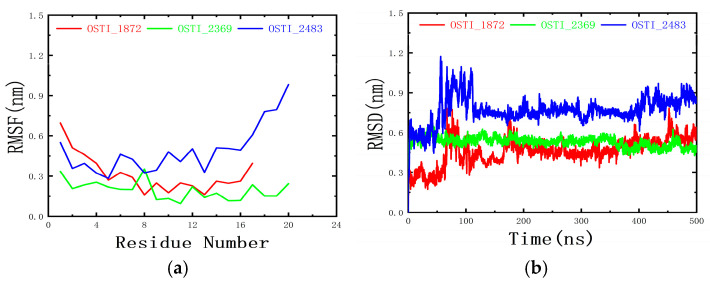
(**a**) RMSF of amino acid residues of peptides; (**b**) RMSD of peptides during molecular dynamics. ns represents the unit of time nanoseconds.

The root mean square fluctuation (RMSF) and root mean square deviation (RMSD) were used to characterise the structural dynamics of the peptides. As shown in Figure 9a, the RMSF profiles of the three peptides exhibited distinct fluctuation patterns. The RMSF values of the residues in OSTI-2483 were mostly above 0.3 nm, indicating that this peptide exhibited greater flexibility and underwent pronounced conformational fluctuations during the MD simulations. In contrast, the RMSF values of most residues in OSTI-1872 were around 0.3 nm, suggesting lower flexibility than that observed for OSTI-2483. The RMSF values of the residues in OSTI-2369 were generally below 0.3 nm, reflecting reduced flexibility and a more stable conformation throughout the simulation.

As shown in Figure 9b, the RMSDs of the three peptides fluctuated markedly during the first 100 ns of the simulations. This initial fluctuation can be attributed to the gradual approach and binding of the peptides to the membrane surface, accompanied by continuous conformational rearrangements to reach an optimal binding state. The RMSD of OSTI-2369 reached equilibrium after approximately 100 ns and stabilised at around 0.48 nm, suggesting that this peptide rapidly achieved equilibrium upon binding to the mixed membrane. In contrast, the RMSDs of the other two peptides stabilised only after approximately 200 ns, indicating that their binding processes required a longer equilibration period.

#### 3.10.3. Analysis of Rg and SASA

**Figure 10:**
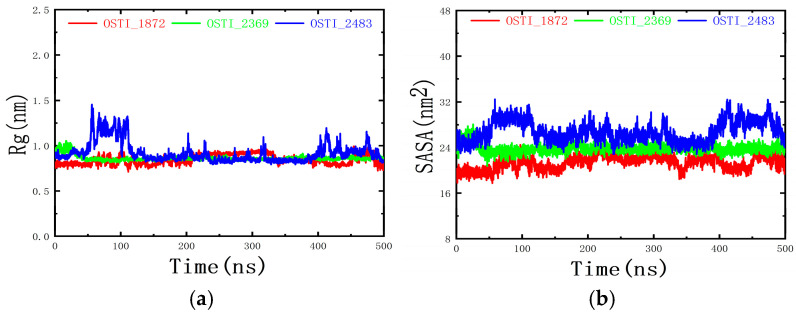
(**a**) Changes in Rg of peptides and (**b**) changes in SASA of peptides during molecular dynamics. ns represents the unit of time nanoseconds.

The radius of gyration (Rg) and solvent-accessible surface area (SASA) were analysed to assess the compactness and structural stability of the antimicrobial peptides during the MD simulations. As shown in Figure 10, the Rg and SASA values of the three peptides fluctuated moderately throughout the simulations without exhibiting any consistent upward or downward trend. These findings indicate that, although minor conformational rearrangements occurred, the overall structural compactness of the peptides remained essentially stable during the dynamic process.

#### 3.10.4. Interaction Energy and Binding Free Energy Analysis

**Figure 11:**
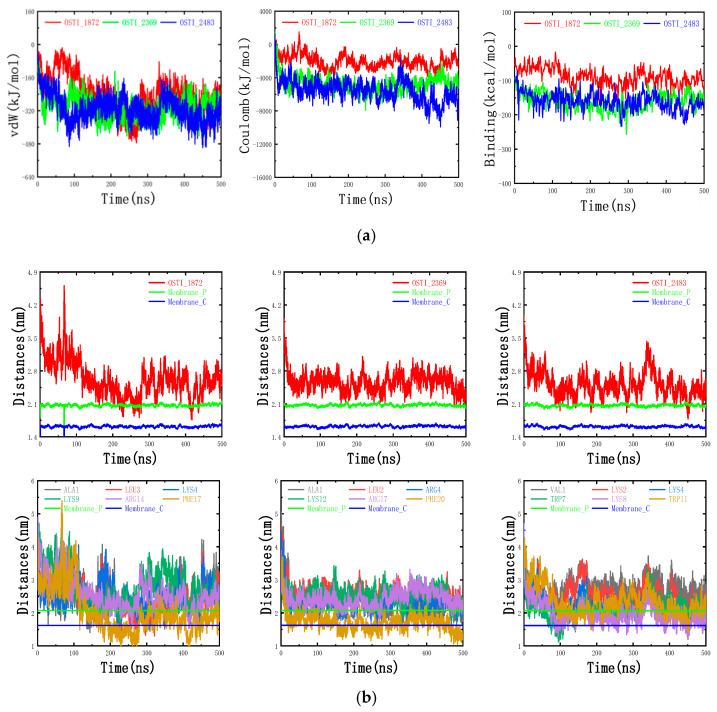
(**a**) Van der Waals interaction energies (vdW), electrostatic interaction energies (Coulomb), and binding free energies between the antimicrobial peptides and the cell membrane. (**b**) Centre-of-mass distances, and the top three polar (charged) and nonpolar amino acid residues with the highest free binding energies between the antimicrobial peptides and the cell membrane mixture. Note: Membrane_P represents the plane of the phospholipid phosphorus atom, and Membrane_C represents the plane of the carbonyl carbon atom of the phospholipid. ns represents the unit of time nanoseconds.

The van der Waals (vdW) and electrostatic (Coulomb) interaction energies between the peptides and the mixed-cell membrane were evaluated throughout the molecular dynamics (MD) simulations to elucidate the driving forces underlying peptide–membrane binding. As shown in Figure 11a, the vdW and electrostatic interaction (Coulomb) energies between OSTI-1872 and the mixed-cell membrane are approximately −240 kJ/mol and −2000 kJ/mol, respectively. For OSTI-2369, the corresponding values are approximately −280 kJ/mol and −4200 kJ/mol, while for OSTI-2483, they are approximately −320 kJ/mol and –6400 kJ/mol. These results indicate that electrostatic forces predominated in mediating the interactions between the peptides and the mixed-cell membrane. The results of the binding free energies via the MM/PBSA method display a trend consistent with that of the electrostatic interaction energies: the binding free energy of OSTI-1872, OSTI-2369, OSTI-2483 is approximately −100 kcal/mol, −149 kcal/mol, and −170 kcal/mol, respectively. Overall, analysis of the interaction and binding free energies indicates that all three antimicrobial peptides exhibit favourable affinities toward the mixed-cell membrane, with OSTI-2483 showing the strongest binding interactions, followed by OSTI-2369 and then OSTI-1872.

The distances between these AMPs and the centroid of the mixed-cell membrane were computed to more intuitively analyse the adsorption and insertion behaviours of these AMPs on the cell membrane surface, as shown in Figure 11b. As shown, the distances between the three AMPs and the membrane centroid are mostly within 2.1–2.8 nm, above the plane of the phosphorus atoms of the phospholipid molecules, indicating that most amino acid residues of the AMPs are bound to the surface of the mixed-cell membrane. In some cases, the distances between the AMPs and the membrane centroid are less than 2.1 nm, suggesting that certain residues penetrate the membrane interior. As can be seen, the polar (charged) residues of the AMPs mainly interact with the hydrophilic head region of the mixed cell membrane, and their centre-of-mass distances are generally above the plane of the carbonyl carbon atoms of the phospholipid molecules. Consistently, analysis of the total interaction energies and binding free energies further demonstrated that the association of the antimicrobial peptides with the membrane was energetically favourable.

## 4. Discussion

The urgent need for substitute antimicrobial therapeutic agents to treat superbugs with antibiotic resistance has been recognised [21]. Scientists have long believed that it is unlikely microbes will develop resistance against AMPs because they attack multiple low-affinity targets, like bacterial membranes, instead of a single high-affinity target [22,23,24]. Many AMPs that have been tested clinically are only suitable for topical application because of their susceptibility to protease degradation, rapid kidney clearance, and systemic toxicity [25,26,27,28]. In this research, a novel skin-derived BBI peptide, named OSTI-1872, was obtained from the skin of the frog *Odorrana schmackeri*. This peptide displayed a potent inhibitory activity against trypsin and weak antimicrobial activities against some Gram-negative bacteria, which is consistent with previous reports of this peptide class [7,29]. Of interest is that this peptide showed a more sensitive antimicrobial effect on drug-resistant bacterial strains of *E. coli* (BAA 2340 and NCTC 13846) than on a non-drug resistant bacterial strain of *E. coli* (ATCC CRM 8739). Therefore, optimising the antimicrobial activity of the parent BBI peptide to build a bifunctional peptide with both potent trypsin-inhibitory activity and strong antimicrobial activity might offer a possibility for new antimicrobial agents against drug-resistant bacteria and a solution to the degradation of antimicrobial peptides by trypsin or trypsin-like proteases [15].

The strategies of structural modification of AMPs revolve around their amino acid sequences. Firstly, possession of several positively charged amino acids is a typical characteristic of AMPs, causing net charges ranging from +2 to +13. Numerous researchers have proven that there is a strong association between charge and antimicrobial activity of many AMPs [30,31,32,33,34]. Another property possessed by AMPs is amphipathicity, which can be realised in many conformations of the peptide, such as the α-helix or β-sheet [8,9]. Positive charges allow peptides to bind to the negatively charged substances on the bacterial membrane surfaces more efficiently; meanwhile, the intense hydrophobic face enables peptides to interact more easily with the bilayer and contribute to the formation of transmembrane pores [35]. Therefore, several bifunctional analogues were designed using rational structural modifications. In one of the analogues, OSTI-2369, an artificial α-helix structure, was added to the N-terminal of the TIL of BBI peptides to increase their amphipathicity [36,37]. For OSTI-2483, the artificial β sheet structure was added to the N-terminal of the trypsin-inhibitory loop, and three lysines were used to substitute for other amino acids to enhance the positive charge of peptides at the flexible 11, 14, and 17 positions, which were considered to have little influence on the inhibitory activity of BBI peptides. OSTI-2380 was designed to remove or decrease trypsin-inhibitory activity via disulfide bridge fracture to examine the impact of this on antimicrobial activity.

The amphipathicity of the parent peptide and its analogues was quantitatively evaluated by calculating the hydrophobic moment. The parent peptide displayed a low hydrophobic moment (μH = 0.176), while the three modified peptides exhibited values of 0.321, 0.135, and 0.179, respectively. These values indicate low-to-moderate amphipathic character and are substantially lower than those typically reported for strongly pore-forming antimicrobial peptides. Importantly, no clear correlation was observed between the hydrophobic moment and the MIC values, suggesting that increased amphipathicity is not the primary determinant of antimicrobial activity in these peptides. Consistent with this observation, only very weak membrane-disruptive activity was detected at their MICs, and no experimental evidence supporting stable pore formation was obtained, which indicates that the peptides do not primarily act through a classical pore-forming mechanism.

CD spectroscopy was employed to experimentally characterise the secondary structure of OSTI-1872 and its analogues under aqueous (10 mM NH_4_Ac) and membrane-mimetic (50% TFE/NH_4_Ac) conditions. In aqueous buffer, all peptides exhibited relatively low α-helical content, indicating predominantly disordered or flexible conformations in solution. Upon exposure to the membrane-mimetic environment, a pronounced increase in α-helical content was observed for OSTI-1872 and OSTI-2369, with α-helix proportions increasing from approximately 19% to over 40%. This environment-induced helix formation is a characteristic feature of many antimicrobial peptides and supports their ability to undergo conformational transitions upon membrane interaction [38]. In contrast, OSTI-2483 displayed a reduced α-helical content in 50% TFE, accompanied by a higher proportion of β-strand structures, suggesting sequence-dependent differences in structural responsiveness and conformational preferences. These CD results provide experimental evidence that the peptides do not adopt a single rigid secondary structure but instead populate multiple conformational states depending on their surrounding environment. When considered together with AlphaFold predictions, this indicates intrinsic tendencies toward secondary structure formation and highlights the intrinsic conformational flexibility of the peptide chains. Such flexibility may allow the peptides to remain structurally dynamic in aqueous environments while undergoing partial folding into ordered conformations upon membrane contact, thereby facilitating adaptive interactions with microbial membranes and contributing to their biological activity.

By comparing OSTI-1872 with the reported OSTI, the residue at the P14 position of the amino acid sequence seems to have a great impact on trypsin-inhibitory activity, as OSTI, with lysine at the P14 position, displayed much better trypsin-inhibitory activity than the residue-14 arginine-substituted in OSTI-1872. In addition, all analogues showed potent anti-trypsin activity, especially OSTI-2369, which demonstrated that the α-helix combined with the conserved loop of BBI peptides did not change its trypsin-inhibitory activity. However, OSTI-2483 displayed a 6-fold decreased Ki value (approx.) compared with the parent peptide. It seems the three substitutions of lysine residues play a negative role in the trypsin-inhibitory activity of BBI peptides. OSTI-2380 displayed the lowest trypsin-inhibitory activity among the analogues designed, which proved that the disulfide bridge between two cysteines plays a significant part in trypsin-inhibitory activity [37].

The results of screening the antibacterial effects of the modified peptides illustrated that the cationic improvement and secondary structure of α helix or β sheet did contribute to enhancing the antimicrobial activity of this BBI peptide. The enhanced antimicrobial activity of analogues against three *E. coli* strains, but not against *A. baumannii*, *S. aureus*, or *E. faecalis*, highlights an intriguing species-dependent selectivity. This behaviour may be partially explained by differences in bacterial envelope architecture and membrane composition. *E. coli*, a Gram-negative bacterium, possesses an outer membrane enriched in negatively charged lipopolysaccharides and phospholipids, which may favour the initial electrostatic association of cationic peptides that primarily act at the membrane surface. In contrast, Gram-positive bacteria such as *S. aureus* and *E. faecalis* lack an outer membrane but contain a thick peptidoglycan layer enriched with teichoic acids, which can impede access of peptides with limited membrane-disruptive capacity. These structural and compositional differences may underlie the observed selective enhancement of antimicrobial activity and further emphasise that the antibacterial efficacy of non-lytic peptides is highly dependent on species-specific membrane properties.

Compared to the negligible activity against all tested microorganisms of parent peptide OSTI-1872, the analogues displayed a pronounced preference for Gram-negative bacteria, particularly *E. coli* strains and *P. aeruginosa*. Increased α-helical propensity in membrane-mimetic environments correlated with enhanced antimicrobial potency; however, peptides retaining partial non-helical conformations remained antimicrobial activity, indicating that complete helix formation is not a prerequisite for bactericidal activity. In addition to α-helicity, increased β-strand content was also associated with improved antimicrobial activity. CD spectroscopy revealed that the more potent analogues exhibited significantly higher β-strand proportions, particularly under membrane-mimetic conditions, suggesting that β-structured regions may facilitate membrane surface adsorption and perturbation. Notably, the analogues also exhibited potent bactericidal effects against MRSA, but remained inactive against non-resistant *S. aureus*, highlighting that rational sequence modification successfully expanded the antibacterial spectrum of the OSTI-derived analogues to include clinically relevant Gram-positive resistant pathogens. This selective activity suggests that peptide efficacy is influenced not only by bacterial Gram classification but also by strain-specific membrane properties.

The time-killing assay compared the bactericidal kinetics of OSTI-1872 and its analogues and revealed distinct, strain-dependent bactericidal kinetics. OSTI-2369 rapidly eliminated *E. coli* NCTC 13846 within 10 min but failed to achieve complete killing of *E. coli* BAA 2340 at MIC within 360 min, suggesting that bacterial surface properties may modulate peptide susceptibility. OSTI-2483 exhibited rapid bactericidal activity against both strains at MIC, indicating a more robust membrane interaction. Meanwhile, all analogues displayed immediate bactericidal effects at 2 × MIC and 4 × MIC regardless of strain, which is much different from the classical AMPs [39], implying that once a critical surface concentration is reached, bacterial killing becomes rapid and less dependent on membrane composition. In general, these bifunctional peptides displayed a faster antibacterial effect which may be caused by multiple factors. In contrast, the analogue of OSTI-2380, with a lower trypsin-inhibitory activity, showed much slower bacteria killing than OSTI-2483, which indicates that trypsin-inhibitory activity does contribute to the properties affecting kinetic antimicrobial activity.

The permeability assay aimed to investigate the antibacterial mechanism of the parent peptide and its analogues. Despite rapid bactericidal activity, all the analogues showed a small increase in permeability compared to the parent peptide, OSTI-1872, whereas they still retained a much lower permeability rate of less than 10% at MIC except OSTI-2380, this observation argues against stable pore formation as the primary killing mechanism. Consistently, molecular dynamics simulations showed that all peptides predominantly embed on the surface of the POPE/POPG mixed membrane and only limited residue insertion into the membrane interior. Together, these findings support a surface adsorption mechanism involving transient membrane perturbation rather than extensive membrane rupture. Interestingly, OSTI-2380, which lacks a disulfide bridge and exhibits reduced trypsin-inhibitory activity, induced higher instantaneous membrane permeability but displayed slower bactericidal kinetics. This finding indicates that excessive conformational flexibility may enhance membrane disruption while compromising killing efficiency, underscoring the role of the conserved disulfide bridge as a structural constraint that balances antimicrobial activity with preservation of trypsin-inhibitory function.

AMPs are widely recognised for their non-receptor-mediated bactericidal activity involving membrane lysis; their primary antibacterial activity is associated with the membrane-lytic mechanism, which directly disrupts the integrity of the bacterial cell membrane and cell wall [40,41,42]. Antimicrobial peptides carry positive charges which attract them to the negatively charged cell membrane. They are also amphiphilic, which can assist in forming pores or in aligning parallel to the surface of the cell membrane. The studied peptides eventually caused damage to the cell membrane of all tested bacteria strains [43,44]. Meanwhile, studies have shown that membrane disturbance is also part of the mechanisms of action that trypsin inhibitors utilise against *S. aureus*—a Gram-positive bacterium. One study found that a trypsin inhibitor was able to cause membrane damage, resulting in cell lysis and an increased production of reactive oxygen species (ROS) [45], while another showed that nucleic acids were released as trypsin inhibitors disrupted membrane integrity [46]. A trypsin inhibitor could also trigger cell death by interacting with and breaking the cytoplasmic membrane [47]. In this study, these modified peptides displayed little permeability activity at their MICs; their antimicrobial activity is not just based on mechanical damage of bacterial membranes but is probably caused by multiple factors, as one study discovered that the antimicrobial activity of the trypsin inhibitor was qualified by the inhibition of the endogenous proteases isolated from the bacteria themselves [48]. It was noticed here that OSTI-2380, with a lower trypsin-inhibitory activity, showed a better permeability effect than OSTI-2483 at the tested concentrations, which indicated that when the trypsin-inhibitory activity of bifunctional peptides decreases, the permeability rate increases, indicating a negative correlation between trypsin-inhibitory activity and permeability enhancement.

Analysis of peptide conformational dynamics further highlighted their structure–function relationships. OSTI-2369 exhibited lower residue flexibility and reached conformational equilibrium more rapidly upon membrane binding, whereas OSTI-2483 displayed higher flexibility accompanied by stronger electrostatic interactions and more favourable binding free energies. This suggests that an optimal balance between structural stability and flexibility governs antibacterial efficiency and strain selectivity. Electrostatic interactions were the dominant driving force for peptide–membrane association, with Coulombic interaction energies increasing progressively from OSTI-1872 to OSTI-2483, in agreement with their antimicrobial potency.

The safety of these peptides was assessed by haemolysis and MTT cell proliferation assays. It is well-known that AMPs can interact with and disrupt the membranes of eukaryotic cells, especially erythrocytes, leading to cytotoxicity, particularly haemolysis. The ability of AMPs to target membranes makes their selectivity low, such that AMPs can attack host cells, resulting in cytotoxicity and haemolytic activity [14]. Interestingly, like the parent peptide, the modified peptides also showed low haemolysis of mammalian erythrocytes and a low antiproliferative activity on normal HaCaT cells. The kinds of structural modifications employed seem to produce BBI peptides, which avoid the problems of the high cytotoxicity characteristic of many AMPs, which plays a large part in preventing these from being taken seriously as new antimicrobial agents in the clinic.

During salt ion and horse serum assays, the peptides maintained stable antibacterial effects, but they all experienced a decrease when treated with CaCl_2_. Free ions with a single positive charge can block AMPs from binding to the bacterial membrane because of the charge repulsion effect; ions with multiple positive charges exert the ability to enhance the rigidity of the bacterial membrane in a manner requiring binding to the phosphate groups on the surface of the membrane with negative charges, thus preventing the formation of pores to reduce the antimicrobial action of AMPs [49,50].

Trypsin was the protease selected to assess the stability of the antimicrobial activity of these peptides, and even though the ratio of peptide to trypsin approached 2:1, the results showed that the antimicrobial activity of OSTI-2369 and OSTI-2483 was retained after incubation with trypsin, whereas the analogue OSTI-2380, which had a lower anti-trypsin activity, showed decreased antimicrobial activity. These results show that these bifunctional peptides possess a degree of resistance to degradation by trypsin, which perhaps offers the possibility of the development of oral antimicrobial peptide formulations. In physiological environments, proteolytic degradation represents a major limitation for the therapeutic application of antimicrobial peptides, as they can be inactivated rapidly by host- or pathogen-derived serine proteases. Unlike conventional strategies that rely on extensive chemical modifications to enhance protease resistance, the dual functionality of BBI-derived peptides integrates antimicrobial activity and protease inhibition within a single molecular scaffold. This built-in protective mechanism may help preserve antibacterial efficacy without compromising membrane interactions or biocompatibility. The intrinsic trypsin-inhibitory activity of OSTI-1872 and its analogues therefore represents a potentially important functional advantage. By partially suppressing trypsin-like protease activity, these peptides may reduce their own proteolytic degradation, thereby prolonging their local persistence and effective antimicrobial action under biologically relevant conditions. Collectively, these features suggest that BBI-based peptides offer a promising alternative design strategy for developing antimicrobial agents with improved stability and sustained activity in protease-rich environments.

Molecular dynamic (MD) simulations showed that the antimicrobial peptides (AMPs) were stably adsorbed onto the mixed POPE/POPG membrane, with several amino acid residues of each peptide partially embedded in the bilayer. The calculated distances between the peptides and the membrane centroid remained within 2.1–2.8 nm for most of the simulation period, positioned above the plane defined by the phosphorus atoms of the phospholipid headgroups. These results indicate that all three AMPs can form stable associations with the mixed membrane. This finding suggests that the majority of amino acid residues in the three AMPs were primarily bound to the membrane surface.

Further analysis of the distances between selected polar (charged) and nonpolar residues and the membrane centroid revealed that the polar (charged) residues were mainly associated with the hydrophilic head region of the membrane. Their distances generally remained above the plane containing the carbonyl carbon atoms of the phospholipids. In contrast, the nonpolar residues in OSTI-1872 and OSTI-2369 penetrated more deeply into the hydrophobic region, with centre-of-mass distances smaller than the carbonyl plane (approximately 1.6 nm), indicating interactions with the acyl chains of the phospholipids. For OSTI-2483, the nonpolar residues also transiently inserted below the carbonyl plane, suggesting that this peptide could embed more deeply for limited periods.

To further evaluate the peptide–membrane interactions, both van der Waals (vdW) and electrostatic interaction energies were calculated. The vdW and electrostatic interaction energies between OSTI-2483 and the mixed membrane were approximately −320 kJ/mol and −6400 kJ/mol, respectively; those for OSTI-2369 were approximately −280 kJ/mol and −4200 kJ/mol; and those for OSTI-1872 were approximately −240 kJ/mol and −2000 kJ/mol. Binding free energies, calculated using the MM/PBSA method, followed the same trend as the interaction energies: approximately −170 kcal/mol for OSTI-2483, −149 kcal/mol for OSTI-2369, and −100 kcal/mol for OSTI-1872. These results indicate favourable binding affinities of all three peptides toward the mixed membrane. Importantly, the electrostatic interaction energies, reaching several thousand kilojoules per mole, were an order of magnitude stronger than the van der Waals contributions, demonstrating that electrostatic forces dominate the peptide–membrane association process. Decomposition of the binding free energies identified key residues contributing most to membrane binding. In OSTI-1872, ALA1, LYS4, LYS9, ARG14, and PHE17 contributed most significantly; in OSTI-2369, LYS3, ARG4, LYS12, and ARG17 were dominant; and in OSTI-2483, LYS2, LYS4, LYS8, and LYS12 made the highest contributions. These key residues were primarily positively charged residues, consistent with the interaction energy analysis. These findings indicate a strong correlation between the number and spatial distribution of positively charged amino acids and the antimicrobial activity of these peptides.

In conclusion, this study demonstrates that rational modification of a natural Bowman–Birk inhibitor peptide can successfully generate bifunctional peptides that combine potent antimicrobial activity with preserved trypsin-inhibitory function. The main novelty of this work lies in the transformation of a non-antimicrobial protease inhibitor peptide into effective antibacterial agents through minimal sequence modifications, without relying on classical pore-forming mechanisms. By integrating antimicrobial assays, membrane permeabilisation studies, and molecular dynamics simulations, a non-lytic, surface-adsorption-dominated mode of action driven primarily by electrostatic interactions with bacterial membranes was revealed. Future studies will focus on elucidating the precise intracellular or membrane-associated targets involved in bacterial killing, evaluating the in vivo efficacy and pharmacokinetics of these peptides, and further optimising the balance between antimicrobial potency, protease inhibition, and stability. Together, these efforts may facilitate the development of BBI-derived peptides as a new class of multifunctional antimicrobial therapeutics.

## 5. Conclusions

In summary, a novel peptide named OSTI-1872, which belongs to the BBI family, was obtained from the skin secretion of *Odorrana schmackeri*. It exhibited strong trypsin-inhibitory activity and weak antimicrobial activity but showed more sensitivity to drug-resistant bacterial strains. Three analogues were subsequently designed to optimise the antimicrobial activity of OSTI-1872 by structural modification. Better antimicrobial activity with potent trypsin-inhibitory activity was observed for the analogues OSTI-2369, OSTI-2483, and OSTI-2380. Meanwhile, the parent peptide and its analogues showed an excellent safety evaluation when tested for haemolytic activity and in a cell proliferation assay using HaCaT cells. Regarding environmental sensitivity, the tested peptides displayed a high degree of stability with salt ions, serum, and trypsin. These data demonstrate with high probability that these analogues could be used as potential antibacterial agents in a clinical setting, and strategies related to the structural modification of BBI peptides may provide another way to develop new antimicrobial drugs. Molecular dynamics simulations showed that all three antimicrobial peptides were stably adsorbed onto the mixed POPE/POPG membrane; they were primarily associated with the membrane surface, with most amino acid residues located above the plane defined by the phosphorus atoms of the phospholipid headgroups. The peptide–membrane association was predominantly governed by electrostatic interactions, which may underlie their potent antimicrobial activity and limited cytotoxicity.

## Figures and Tables

**Table 1:** The sequence of BBI peptides and their physicochemical properties.

Peptides	Sequences	Hydrophilicity	Hydrophobic Moment	Net Charge
OSTI [16]	AALKGCWTKSIPPKPCF-NH_2_	–0.21	0.177	3.91
OSTI-1872	AALKGCWTKSIPPRPCF-NH_2_	–0.21	0.176	3.91
OSTI-2369	ALKRALKRCWTKSIPPRPCF-NH_2_	0.18	0.321	6.91
OSTI-2483	VKWKVKWKCWTKSKPPKPCK-NH_2_	0.43	0.135	8.91
OSTI-2380	VKWKVKWKCWTKSKPPKPK-NH_2_	0.51	0.179	8.95

**Table 2:** Inhibitory activity of OSTI-1872 and its analogues against trypsin.

Peptides	Sequences	Trypsin Ki (μM)
OSTI-1872	AALKGCWTKSIPPRPCF-NH_2_	0.023
OSTI-2369	ALKRALKRCWTKSIPPRPCF-NH_2_	0.044
OSTI-2483	VKWKVKWKCWTKSKPPKPCK-NH_2_	0.131
OSTI-2380	VKWKVKWKCWTKSKPPKPK-NH_2_	8.779

**Table 3:** The MICs/MBCs (μM) of peptides against selected microorganisms.

Bacteria Strains		OSTI-1872	OSTI-2369	OSTI-2483	OSTI-2380
Gram-negative bacteria	*E. coli* (ATCC CRM 8739)	128/256	8/8	4/4	4/4
	*E. coli* (BAA 2340)	128/256	8/8	8/8	4/8
	*E. coli* (NCTC 13846)	128/256	8/8	4/4	4/8
	*P. aeruginosa* (ATCC CRM 9027)	256/512	16/16	8/16	16/16
	*K. pneumoniae* (ATCC CRM 43861)	>512	32/32	64/64	64/128
	*A. baumannii* (BAA 747)	>512	256/256	128/128	>512
Gram-positive bacteria	*S. aureus* (ATCC CRM 6538)	>512	>512	512/512	>512
	*E. faecium* (NCTC 12697)	>512	>512	>512	>512
	MRSA (NCTC 12493)	>512	16/16	8/8	16/16
Yeast	*C. albicans* (ATCC 10231)	>512	>512	>512	>512

**Table 4:** The values of MIC/MBC (μM) against *E. coli* (ATCC CRM 8739) after peptides were incubated with salt ions and horse serum.

Ionic Liquids	OSTI-1872	OSTI-2369	OSTI-2483	OSTI-2380
1% DMSO	128/256	8/8	4/4	4/4
FeCl_3_	128/256	8/8	4/4	4/8
CaCl_2_	>512	128/128	8/8	32/32
NaCl	>512	4/4	8/8	8/8
KCl	128/256	8/16	4/4	4/4
MgCl_2_	>512	16/16	8/8	8/8
NH_4_Cl	128/256	4/4	4/4	8/8
Serum	128/128	16/16	4/8	4/8

**Table 5:** The values of MIC/MBC (μM) against *E. coli* (ATCC CRM 8739) after peptides were treated with trypsin.

Trypsin	OSTI-1872	OSTI-2369	OSTI-2483	OSTI-2380
Contrast	128/256	8/8	4/4	4/4
0.25 mg/mL	128/256	8/8	4/4	64/>64
0.5 mg/mL	128/256	8/8	8/16	64/>64

## Data Availability

The data supporting the findings of this study are available within the article and its Supplementary Information Files.

## Supplementary Materials

The following supporting information can be downloaded at https://www.mdpi.com/article/10.3390/biom16010148/s1, Figure S1: Purity of (a) OSTI-1872, (b) OSTI-2369, (c) OSTI-2483, and (d) OSTI-2380 was confirmed by RP-HPLC; Figure S2: MALDI-TOF MS spectrometry of the five synthetic peptides: (a) OSTI-1872, (b) OSTI-2369, (c) OSTI-2483, and (d) OSTI-2380.
