# Supplementary material for: Bifunctional Peptides Generated by Optimising the Antimicrobial Activity of a Novel Trypsin-Inhibitory Peptide from Odorrana schmackeri
Source: Biomolecules. 2026 Jan 14;16(1):148. doi: 10.3390/biom16010148 (PMC12838809; doi:10.3390/biom16010148)
Supplement: Supplementary file 1 [file biomolecules-16-00148-s001.zip › biomolecules-4066961-supplementary.pdf]

Supplementary data

Novel Bifunctional Peptides Generated by Optimising the Antimicrobial Activity of a Trypsin Inhibitory Peptide from *Odorrana Schmackeri*

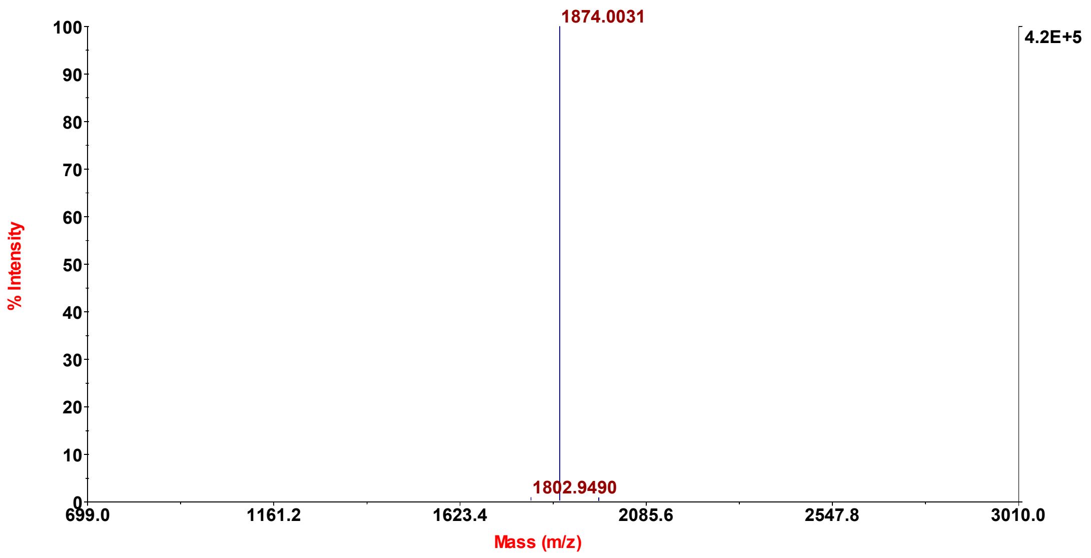

(a)

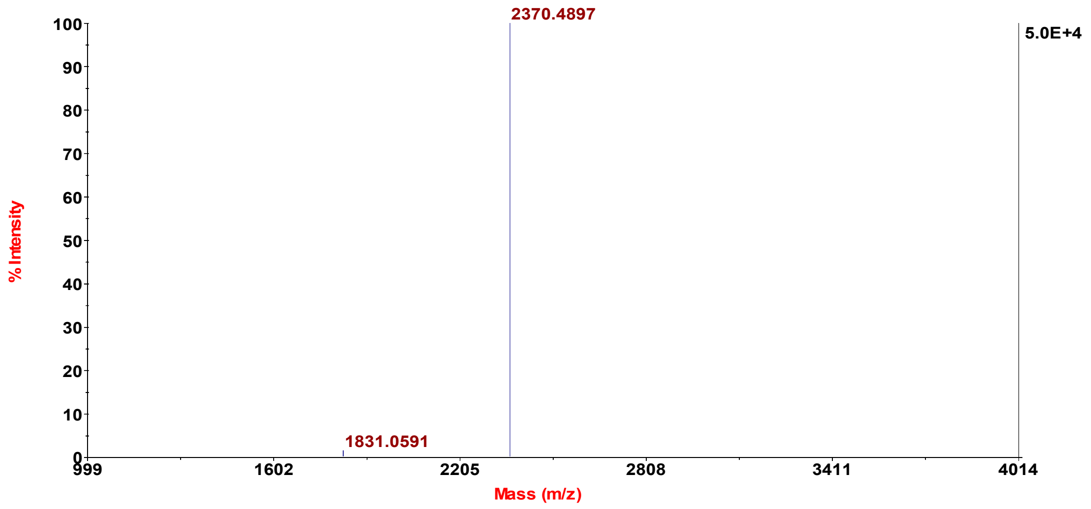

(b)

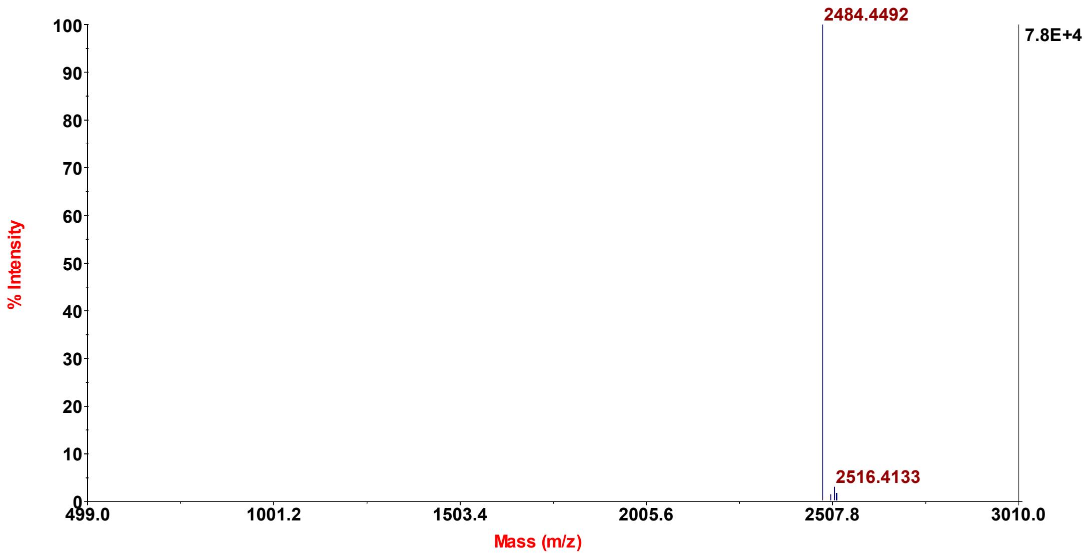

(c)

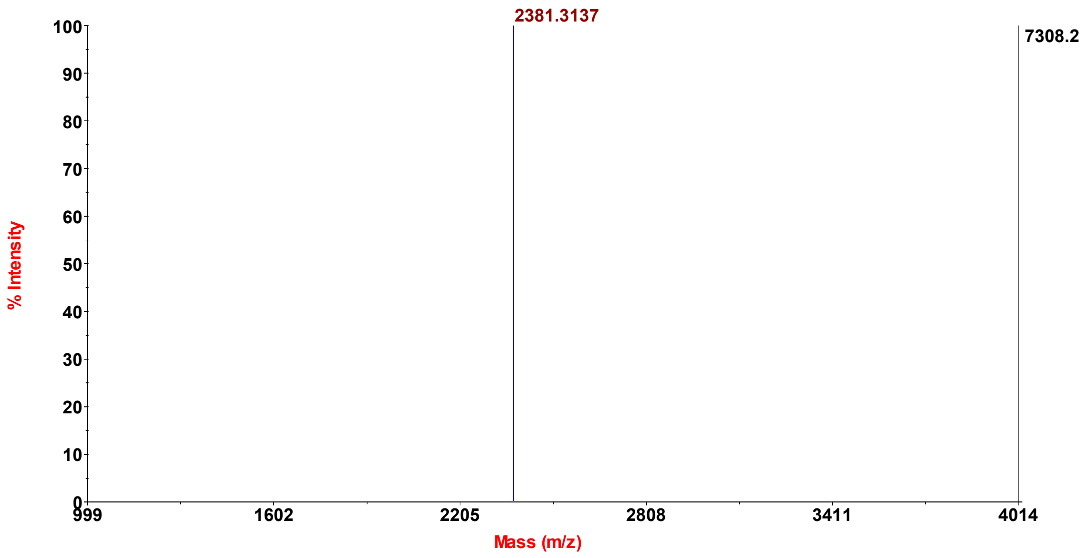

(d)

Figure S1. MALDI-TOF MS spectrum of OSTI-1872 and its analogues (a-d).

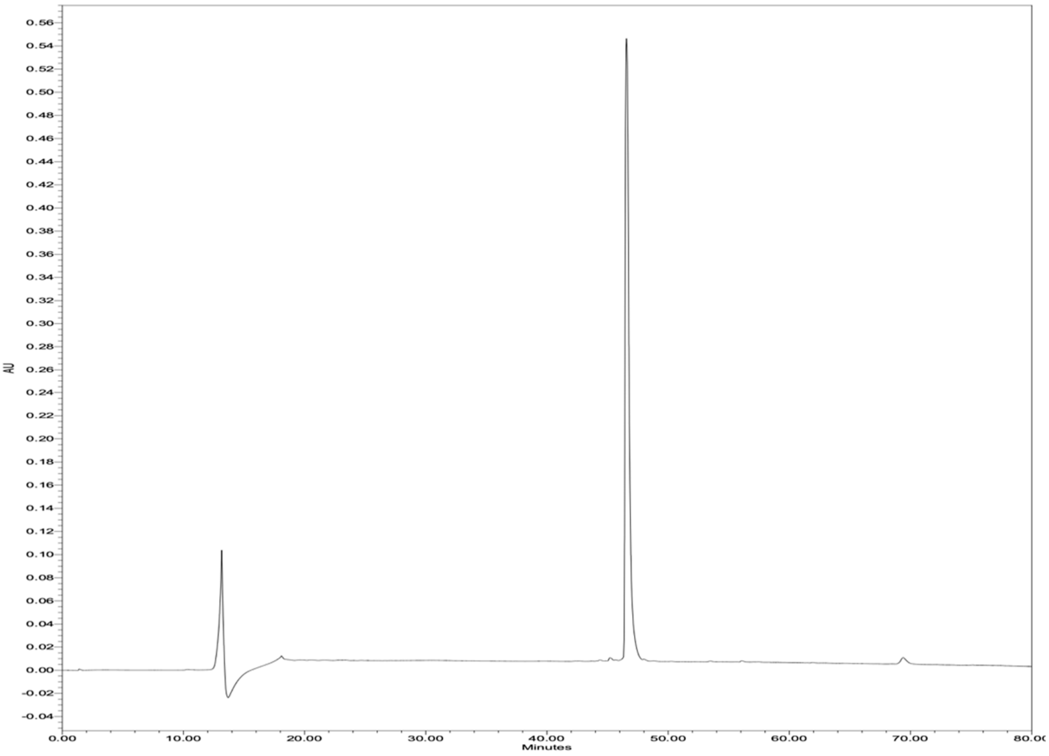
AU
Minutes
0.56
0.54
0.52
0.50
0.48
0.46
0.44
0.42
0.40
0.38
0.36
0.34
0.32
0.30
0.28
0.26
0.24
0.22
0.20
0.18
0.16
0.14
0.12
0.10
0.08
0.06
0.04
0.02
0.00
-0.02
-0.04
0.00
10.00
20.00
30.00
40.00
50.00
60.00
70.00

(a)

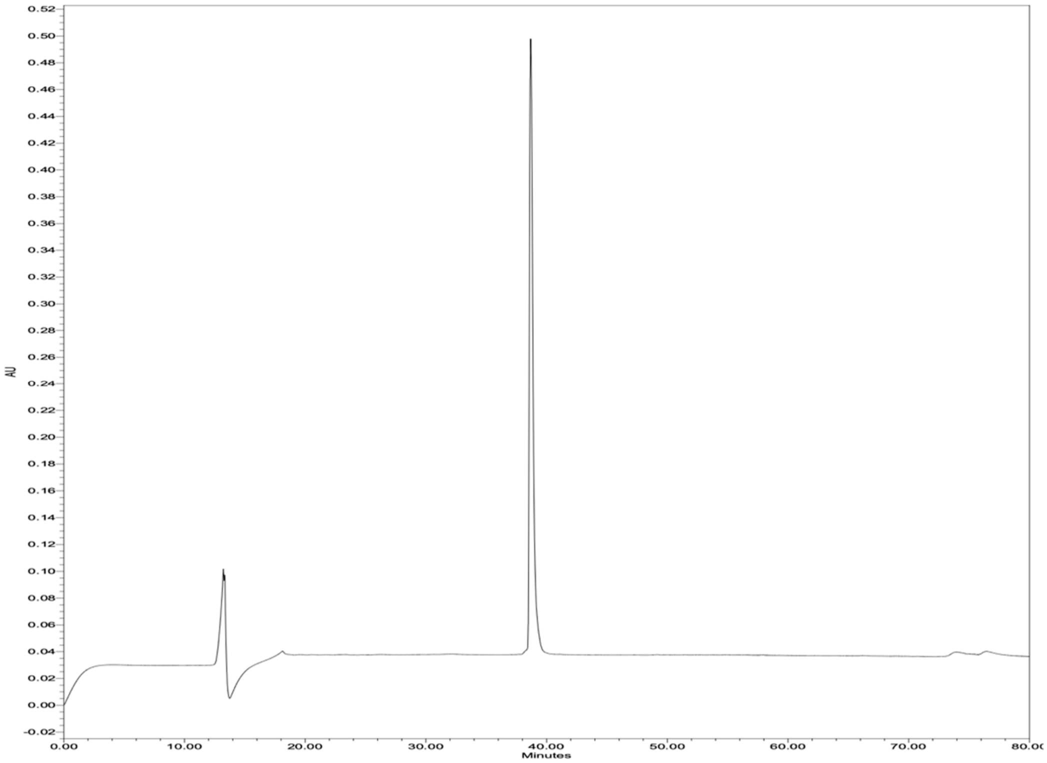
AU
Minutes
0.52
0.50
0.48
0.46
0.44
0.42
0.40
0.38
0.36
0.34
0.32
0.30
0.28
0.26
0.24
0.22
0.20
0.18
0.16
0.14
0.12
0.10
0.08
0.06
0.04
0.02
0.00
-0.02
0.00
10.00
20.00
30.00
40.00
50.00
60.00
70.00

(b)

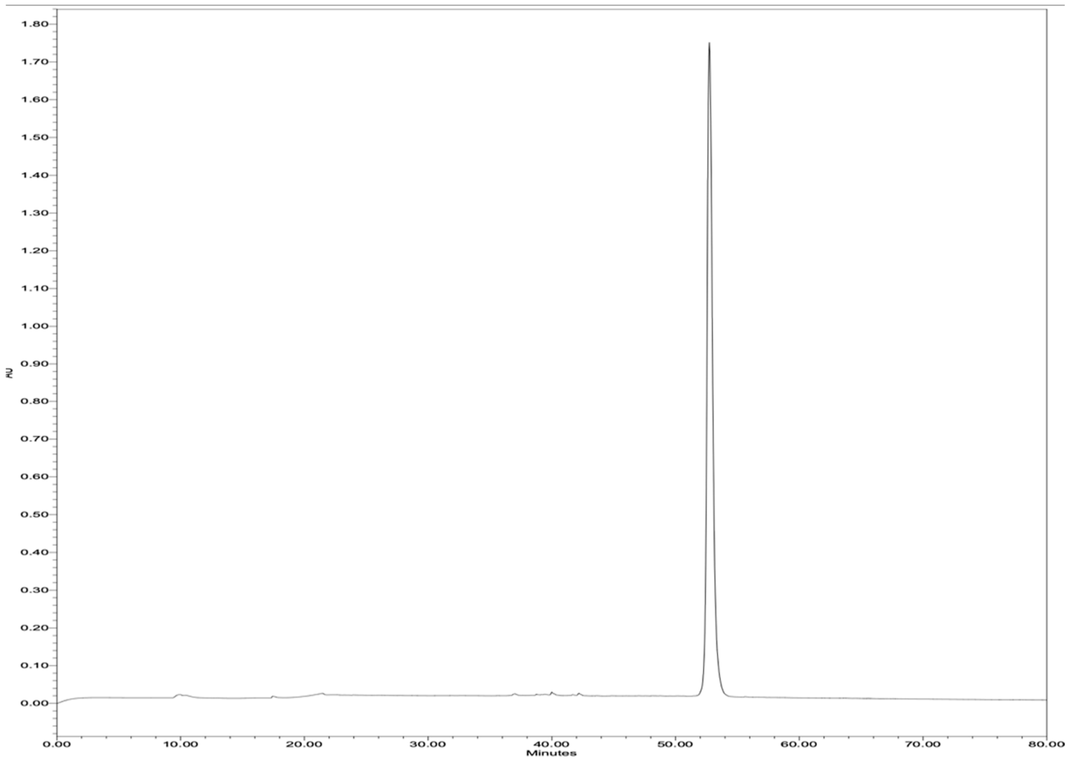

(c)

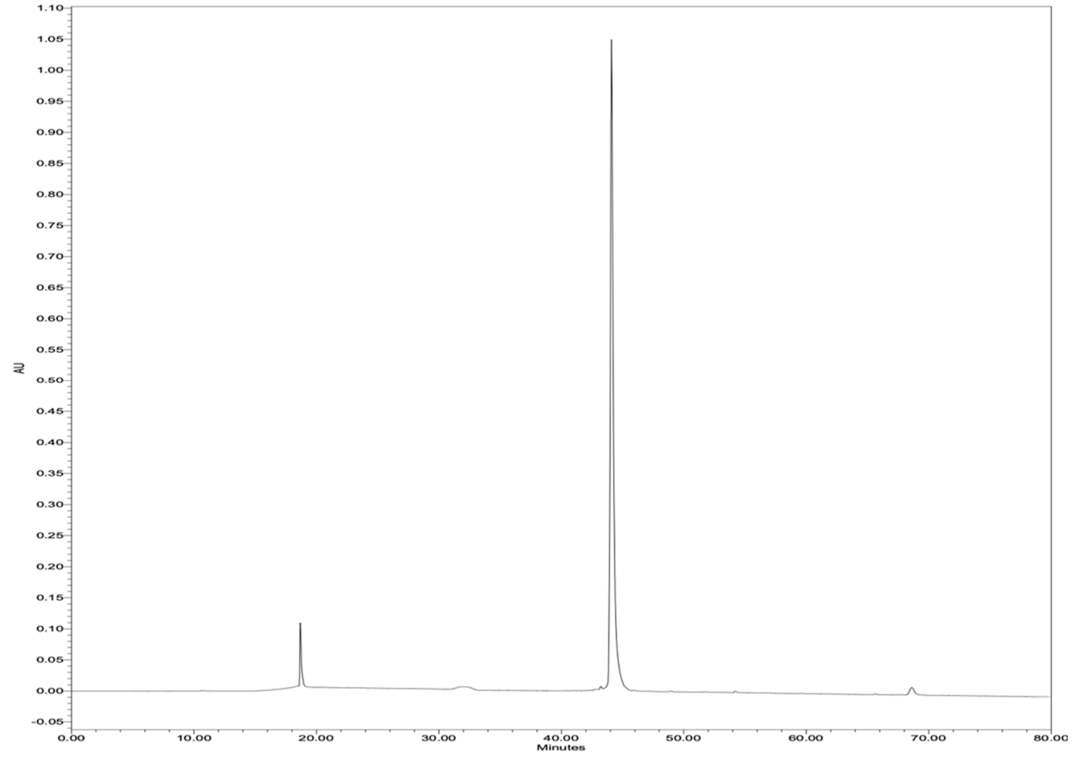

(d)

Figure S2. Purity of OSTI-1872 and its analogues (a-d) was confirmed by Rp-HPLC.
